# Quasi-Power Law Ensembles: Nonextensive Statistics or Superstatistics

**DOI:** 10.3390/e28020171

**Published:** 2026-02-02

**Authors:** Maciej Rybczyński, Grzegorz Wilk, Zbigniew Włodarczyk

**Affiliations:** 1 Institute of Physics, Jan Kochanowski University, 25-406 Kielce, Poland; zbigniew.wlodarczyk@ujk.edu.pl; 2 Department of Fundamental Research, National Center for Nuclear Reasearch, 02-093 Warsaw, Poland

**Keywords:** multiparticle production processes, Tsallis distribution

## Abstract

In phenomenological studies of multiparticle production, transverse-momentum spectra measured in experiments frequently display an approximately power-law falloff, for which the Tsallis-type functional form is commonly employed as an effective parametrization. Within this framework, the emergence of such spectra is interpreted as a manifestation of nonextensive statistical behavior. An analogous power-law structure, however, can be reproduced without explicitly postulating Tsallis statistics by assuming the presence of intrinsic fluctuations of the local temperature (T) in the hadronizing medium; in that case, the observed deviations from a purely exponential spectrum are encapsulated by the nonextensivity index (q). We show that temperature fluctuation mechanisms capable of generating Tsallis-like power-law distributions in multiparticle production necessarily induce nontrivial inter-particle correlations among the emitted hadrons. Building on this observation, we outline a strategy to discriminate fluctuations realized on an event-by-event basis from those arising predominantly through event-to-event variability. Such a separation may be particularly pertinent for the characterization of high-multiplicity (high-density) final states produced at the Large Hadron Collider.

## 1. Introduction

Since the earliest collider measurements, multiparticle production has served as a primary probe of how the energy carried by incident projectiles is redistributed into a large number of final-state hadrons. Quantitative information on this conversion mechanism is extracted from inclusive spectra measured over the kinematically accessible phase space [1]. A standard phenomenological classification separates the produced particles into two broad components. The dominant population resides in the central, high-occupancy domain of relatively low transverse momenta, $p_{T}\leq p_{T_{0}}$, and is typically associated with soft dynamics. A second component occupies the higher-$p_{T}$ tail, $p_{T}\gg p_{T_{0}}$, and is commonly interpreted as originating from hard processes described within quantum chromodynamics (QCD), populating the remaining, less densely filled regions of phase space. The corresponding $p_{T}$ dependences are qualitatively distinct: the soft sector is often approximated by an exponential form, $f\left(p_{T}\right)∼\exp\left(-p_{T}/T\right)$, whereas the hard sector is well represented by a power-law falloff, $f\left(p_{T}\right)∼p_{T}^{-n}$. This dichotomy motivates the use of statistical (thermal-like) descriptions with a scale parameter *T* (“temperature”) for the former and QCD-based partonic scattering dynamics governed by an index *n* for the latter.

A compact parametrization that smoothly covers both limiting behaviors is provided by the Tsallis form arising in nonextensive statistical mechanics [2,3],

(1) $$h\left(E\right)=\frac{2-q}{T}{\left(1+\left(q-1\right)\frac{E}{T}\right)}^{\frac{1}{1-q}},$$

with q=1+1/n. In statistical–mechanical language, *q* is termed the nonextensivity parameter because it quantifies deviations from extensivity. The interpolation property is explicit: as q→1, Equation (1) reduces to the Boltzmann–Gibbs (BG) exponential distribution with *T* assuming the role of temperature, whereas for E/T≫1, it approaches a power law.

Formulas of the quasi-power-law type in Equation (1) are extensively used well beyond multiparticle phenomenology [4]; nevertheless, in high-energy collisions, they have become a standard tool for both data reduction and modeling of inclusive observables (see, e.g., Ref. [5] and references therein).

Historically, the same functional structure was introduced in Refs. [6,7], later reidentified in Ref. [8], and first employed in fits to experimental spectra in Ref. [9]. In this representation, the auxiliary separation scale $p_{T_{0}}$, which artificially partitions “soft” and “hard” regions, is not required. Under the name of a QCD-motivated Hagedorn-like formula, it has since become one of the conventional phenomenological ansätze in $p_{T}$-spectrum analyses.

Unlike many purely empirical fits, the Tsallis framework, interpretable as a generalization of Boltzmann statistics, has a well-developed conceptual motivation. As increasingly precise measurements accumulated, it became apparent that the traditional Boltzmann entropy (in thermal applications) and the Shannon entropy (as an information measure) do not account for data over the full range of observed values. Experimentally, one observes systematic departures from the expected exponential behavior in one context and from Poisson statistics in another [1,10]. Such deviations are usually attributed to additional dynamical ingredients producing correlations and fluctuations not captured by equilibrium thermodynamics or by the standard Shannon measure [11]. This motivates either augmenting the standard entropy maximization procedure by suitable constraints or generalizing the entropy functional itself, so that a modified entropy, together with new parameters, can describe complex systems without auxiliary assumptions.

Consequently, many generalized entropies and information measures have been proposed across scientific disciplines (see, e.g., [3,11,12,13,14,15,16] and references therein), frequently characterized by nonextensivity/nonadditivity (Tsallis entropy $S_{q}$ is nonadditive: $S_{q}\left(AB\right)=S_{q}\left(A\right)+S_{q}\left(B\right)+\left(1-q\right)S_{q}\left(A\right)S_{q}\left(B\right)$, where *A* and *B* are independent in the sense that f(AB)=f(A)f(B), and *q* quantifies the degree of nonadditivity (we assume the same *q* for both subsystems in what follows).). Here, we restrict attention to Tsallis entropy [3] $S_{q}$, which reduces to Boltzmann–Shannon entropy at q=1, i.e., $S=S_{q=1}$:

(2) $$S_{q}=\frac{1-\int dE\;f^{q}\left(E\right)}{q-1}\;\;\;\overset{q\to 1}{⟹}\;-\int dE\;f\left(E\right)\ln f\left(E\right),$$

and which is currently among the most commonly used entropic forms in the particle production context discussed above. Its practical attractiveness stems from the associated quasi-power distribution $f_{q}\left(x\right)$,

(3) $$f_{q}\left(x\right)=\left(2-q\right){\left[1-(1-q)x\right]}^{\frac{1}{1-q}}\;\overset{q\to 1}{⟹}\;f\left(x\right)=\exp\left(-x\right),$$

which, as established in [6,7,8], provides a particularly effective description of diverse observables across their full measured dynamical range. The distribution (1) is obtained by maximizing (2) under appropriate constraints on the admissible f(E). More generally, many nonequilibrium or complex systems fall outside standard equilibrium theory yet are well captured within nonextensive entropy approaches, motivating the use of the index *q* as a compact descriptor of certain aspects of complexity [3].

In practice, however, discussions of “nonextensivity” are often centered on the Tsallis-shaped distributions rather than on the entropy functional itself. Indeed, Tsallis-like quasi-power forms can be generated through multiple mechanisms without explicit reference to entropy [17]. A large class of such mechanisms is naturally organized within superstatistics, i.e., a superposition framework appropriate to driven nonequilibrium systems with stationary states and fluctuations of an intensive parameter [17,18].

In this setting, the exponential law $f\left(E\right)=\frac{1}{T}\exp\left(-E/T\right)$ with scale *T* is generalized by allowing *T* to fluctuate [17]:

(4) $$h\left(E\right)=\int_{0}^{\infty }\exp\left(-E/T\right)\;g\left(1/T\right)\;d\left(1/T\right).$$

If 1/T is gamma distributed,

(5) $$g\;\left(\frac{1}{T}\right)=\frac{1}{\Gamma \;\left(\frac{1}{q-1}\right)}\frac{T_{0}}{q-1}{\left(\frac{1}{q-1}\frac{T_{0}}{T}\right)}^{\frac{2-q}{q-1}}\exp\;\left(-\frac{1}{q-1}\frac{T_{0}}{T}\right),$$

then the marginal spectrum becomes Tsallis [17], $h\left(E\right)=\frac{2-q}{T}{\left[1-\left(1-q\right)\frac{E}{T}\right]}^{\frac{1}{1-q}}$, and the strength of temperature fluctuations is encoded in

(6) $$\omega_{T}^{2}=\frac{\mathrm{Var}(T)}{{\langle T\rangle }^{2}}=q-1.$$

Operationally, the nonextensive character manifests itself through the quasi-power structure of the resulting spectra, i.e., via the appearance of *q* in the Tsallis form. Nevertheless, the parameter *q* may admit multiple physical interpretations because Tsallis-type distributions can be produced by different microscopic scenarios, and in principle can also be derived within Shannon entropy maximization by a judicious choice of constraints.

While nonextensive statistical mechanics has enabled substantial progress in modeling uncertainty and variability in complex systems, a number of conceptual questions remain open. These include, among others, the physical interpretation of *q* and the rationale for modifying the entropy functional [19] (“It is just more likely, that is all. It is a good guess. And we always try to guess the most likely explanation, keeping in the back of the mind the fact that if it does not work we must discuss the other possibilities.” (R.P. Feynmann [20]).).

In this work, we investigate whether the quasi-power behavior observed in inclusive spectra from multiparticle production is more naturally attributed to a superstatistical fluctuation mechanism or to genuinely nonextensive dynamics.

## 2. Some Consequences of Temperature Fluctuations

The Tsallis functional form, considered strictly as a quasi-power-law probability density, can emerge through multiple constructions that do not appeal to any entropy extremization principle [17]. We briefly develop several representative realizations. Although their starting points differ, the resulting Tsallis behavior can typically be recast within a unified superstatistical interpretation, namely, as an effective consequence of fluctuations of an underlying scale parameter.

Differentiating the superstatistical representation, Equation (4), with respect to *E*, gives

(7) $$\frac{dh(E)}{dE}=-\int \frac{1}{T}\exp\;\left(-\frac{E}{T}\right)\;g\;\left(\frac{1}{T}\right)\;d\;\left(\frac{1}{T}\right).$$

An integration by parts yields

(8) $$\frac{dh(E)}{dE}=-\frac{1}{T_{0}+\left(q-1\right)E}\;h\left(E\right),$$

which is mathematically analogous to rate equations encountered in preferential-attachment settings. Interpreted physically, this corresponds to a linear dependence of the effective scale on the variable itself, as may occur when attachment-like correlations are present; in particular, one may implement $T_{0}\to T_{0}+\left(q-1\right)E$. The defining equation of the exponential law is then deformed in a way that produces a Tsallis solution with q>1 [21,22]:

(9) $$\frac{df(E)}{dE}=\frac{f(E)}{T_{0}}\;\to \;\frac{dh(E)}{dE}=\frac{h(E)}{T_{0}+\left(q-1\right)E}\;\Rightarrow \;h\left(E\right)=\frac{2-q}{T_{0}}{\left[1-\left(1-q\right)\frac{E}{T_{0}}\right]}^{\frac{1}{1-q}}.$$

Since Equation (3) provides a satisfactory description across the full measured domain, covering regions often interpreted as thermal as well as those usually associated with hard processes, its origin cannot be uniquely attributed to Tsallis entropy. More broadly, for any target probability law, one may specify (i) a corresponding entropy for which it is the maximizer, and (ii) a set of constraints under which maximization of Shannon entropy reproduces that law [23]. For our purposes, the key point is that if the constraints are chosen to encode the dominant dynamical features of the system, one can, in effect, remain within a Shannon-based formulation [24]. For example, 〈x〉=const leads to an exponential distribution, $\langle x^{2}\rangle =\mathrm{const}$ yields a Gaussian, 〈lnx〉=const generates a gamma distribution, and $\langle \ln\left(1+x^{2}\right)\rangle =\mathrm{const}$ produces a Cauchy law. In general, imposing ∫dxf(x)φ(x)=const leads to an exponential-family solution $f\left(x\right)=\exp[\lambda_{0}+\lambda \varphi \left(x\right)]$, with $\lambda_{0}$ and λ fixed by normalization and the constraint. To obtain Equation (1) via such a Shannon maximization route, one may adopt a constraint of the form

(10) $$\langle \ln\;\left[1-\left(1-q\right)\frac{E}{T}\right]\rangle =\frac{q-1}{2-q}.$$

For completeness, consider the Boltzmann (Shannon) entropy,

(11) S=−∫h(E)lnh(E)dE,

whose extremum condition reads

(12) $$\frac{dS}{d\ln h(E)}=-\int \exp\left[\ln h\left(E\right)\right]\;\ln h\left(E\right)\;dE-\int h\left(E\right)\;dE=-\langle \ln h\left(E\right)\rangle -1=0,$$

i.e., 〈lnh(E)〉=−1. Using Equation (8), one finds

(13) $$d\ln h\left(E\right)=-\frac{1}{q-1}\;d\ln\;\left[T_{0}+\left(q-1\right)E\right],$$

and thus an equivalent statement of the constraint is

(14) $$\frac{1}{q-1}\langle \ln\;\left[T_{0}+\left(q-1\right)E\right]\rangle =1.$$

The prefactor (2−q) in Equation (10) arises solely from the normalization of h(E).

Temperature fluctuations can be re-expressed as fluctuations of the produced multiplicity. In particular, a Negative Binomial form, $P\left(N\right)=\frac{\Gamma (N+k)}{\Gamma (N+1)\Gamma (k)}\;\gamma^{k}{\left(1+\gamma \right)}^{-k-N}$, results when the mean $\bar{N}$ in a Poisson multiplicity distribution, $P\left(N\right)={\bar{N}}^{\;N}\exp\left(-\bar{N}\right)/N!$, is itself randomized according to a gamma distribution:

(15) $$g\left(\bar{N}\right)=\frac{1}{\Gamma (k)}\;\gamma^{k}\;{\bar{N}}^{k-1}\exp\left(-\gamma \bar{N}\right),$$

with $\gamma =k/\langle \bar{N}\rangle$. Here, $\bar{N}$ denotes the eventwise mean multiplicity, whereas 〈N〉 indicates averaging over the event ensemble. Identifying fluctuations of $\bar{N}$ with fluctuations of *T* allows one to restate this result in thermodynamic language. For fixed available energy *U*, one has $T=U/\bar{N}$ and $\langle \bar{N}\rangle =U/\langle T\rangle$, so that $g(\bar{N})$ can be mapped to a distribution for 1/T of the form Equation (5), with the correspondence q−1=1/k.

## 3. Experimental Insight

When Tsallis-type quasi-power-law behavior in multiparticle spectra is generated through fluctuations of the effective scale, the construction necessarily induces correlations among the produced particles. This observation enables, in principle, an empirical test designed to separate fluctuations that occur internally within a single event from those that manifest primarily as event-to-event variability. Such a diagnostic is of particular interest for very-high-multiplicity (high-density) final states accessible at the LHC.

Two experimentally addressable consequences of *q*-based descriptions are especially relevant: correlations implied by the *N*-body Tsallis structure and the pattern of event-resolved fluctuations. The correlation aspect becomes explicit upon considering the *N*-particle Tsallis distribution $h(E_{i=1,\ldots ,N})$. Under Boltzmann–Gibbs assumptions, independent emission implies exact factorization of the joint density into a product of single-particle spectra. In contrast, a simple product of one-particle Tsallis forms does *not* reproduce an *N*-particle Tsallis distribution [25]. Achieving $h(E_{i=1,\ldots ,N})$ requires implementing temperature fluctuations at the level of the *N*-particle BG distribution; the resulting mixture is intrinsically non-factorizable and therefore correlates the particles. The induced energy covariance is

(16) $$Cov\left(E_{i},E_{j}\right)=\frac{T^{2}\left(q-1\right)}{{\left(3-2q\right)}^{2}\left(4-3q\right)}.$$

For event-by-event variability, it is useful to distinguish two limiting possibilities, depending on whether the effective temperature is constant within a given event or fluctuates within the event.

(i) **Pure event-to-event variability.** Each event is characterized by a fixed temperature $T=T_{k}$, but the value changes across events due to differing initial conditions. In this case, within any single event, one expects an exponential spectrum, and Tsallis-like deviations arise only after constructing an inclusive spectrum by averaging over $N_{ev}$ events, $k=1,2,\ldots ,N_{ev}$. The inclusive Tsallis behavior then reflects fluctuations associated with varying initial conditions.
(ii) **Intra-event variability.** The effective temperature fluctuates within a single event around some characteristic scale $T_{0}$. Then, a departure from a purely exponential shape appears already at the single-event level, with the eventwise spectrum described by Equation (1) with q>1. This corresponds to a situation where different subregions or sub-collisions within the same event are characterized by different temperatures (formally consistent with a nonextensive ensemble described by Tsallis entropy) (Unlike pure event-to-event variability, which rules out the possibility of nonextensive statistics, intra-event variability does not. In a single p + p interaction, one would expect fluctuations in the number of particle sources, e.g., quark–gluon strings, which can overlap, forming ropes of different colors, identified as sources with different temperatures.).

As argued in Ref. [26], event-resolved multiparticle data provide a natural laboratory for testing whether a hadronizing source exhibits such temperature variability in a thermodynamic interpretation. Transverse-momentum spectra from Pb + Pb collisions at TeV-scale LHC energies are particularly promising for discriminating scenarios (i) and (ii). The LHC enables p+p and nucleus–nucleus interactions up to $\sqrt{s}=14$ TeV, thereby extending the phase space for fluctuation analyses. In the highest-energy p+p collisions, the charged multiplicity at midrapidity is expected to be only ∼10 for |η|≤0.5, increasing by roughly a factor of five when considering the full rapidity range (ALICE can access approximately −5.0≤η≤3.5, while CMS and ATLAS typically cover |η|≤2.5). The decisive advantage of heavy-ion collisions is the approximately ∼A enhancement of multiplicity; for central Pb+Pb, one expects about ∼2500 particles within the rapidity acceptance. This enables single-event distributions to be studied over roughly three decades in yield, making it feasible to infer the distribution shape within an individual event.

To illustrate what can already be inferred, consider existing RHIC measurements of event-by-event fluctuations of the mean transverse momentum $\bar{p_{T}}$. The STAR detector analyzed Au + Au collisions at $\sqrt{s_{NN}}=20$, 62, 130, and 200 GeV. STAR reports [27] that $Var\left(\bar{p_{T}}\right)/{\langle \bar{p_{T}}\rangle }^{2}≃10^{-4}$, where $\bar{p_{T}}$ denotes the within-event average and $\langle \bar{p_{T}}\rangle$ denotes averaging over the event ensemble. The quoted variance is obtained as $Var\left(\bar{p_{T}}\right)=Var{\left(\bar{p_{T}}\right)}_{\mathrm{data}}-Var{\left(\bar{p_{T}}\right)}_{\mathrm{mixed}}$, with mixed events constructed by randomly selecting one track from events drawn from the same centrality and event-vertex bins. Ten centrality classes and five or ten longitudinal-vertex bins (depending on statistics at a given energy) were used, ensuring that the mixed-event reference preserves the multiplicity distribution.

If one adopts scenario (i) and assumes $\bar{p_{T}}\propto T$, then $Var\left(T\right)/{\langle T\rangle }^{2}=Var\left(\bar{p_{T}}\right)/{\langle \bar{p_{T}}\rangle }^{2}≃10^{-4}$, implying $q-1≃10^{-4}$. This magnitude is markedly smaller than the characteristic $q-1≃10^{-2}$ extracted from inclusive $p_{T}$ fits [28,29,30,31,32]. In contrast, under scenario (ii), where *T* varies across sub-collisions within the same event, the variance of the eventwise mean is reduced by the effective number of sources. For $N_{P}$ projectile participants, one expects $Var\left(\bar{p_{T}}\right)≃Var\left(T\right)/N_{P}$, so that for $N_{P}≃100$, one obtains $Var\left(T\right)/{\langle T\rangle }^{2}≃10^{-2}$, comparable to values inferred from inclusive spectra. Similar reasoning can be applied to PHENIX measurements [33].

The average number of binary nucleon–nucleon collisions, $N_{coll}$, is not directly measured and is typically inferred from modeling, commonly within a Glauber framework [34]. In the standard implementation, $N_{coll}\left(b\right)=\sigma_{NN}\left(s\right)T_{AB}\left(b\right)$, with $\sigma_{NN}\left(s\right)=28.8+0.0458\ln^{2.374}\left(s\right)$ and $T_{AB}\left(b\right)$ the nuclear thickness function at impact parameter *b*.

For central nucleus–nucleus interactions, $N_{coll}$ is large, yet a substantial fraction of collisions occur at comparatively low effective nucleon–nucleon energies, implying a modest charged yield per binary collision. Standard Glauber estimates give roughly 800–1000 collisions in the SPS-RHIC energy domain, whereas modified Glauber approaches that incorporate energy conservation yield smaller values of about 500–600; enforcing energy conservation can therefore significantly reduce $N_{coll}$ relative to standard estimates [35].

**Figure 1:**
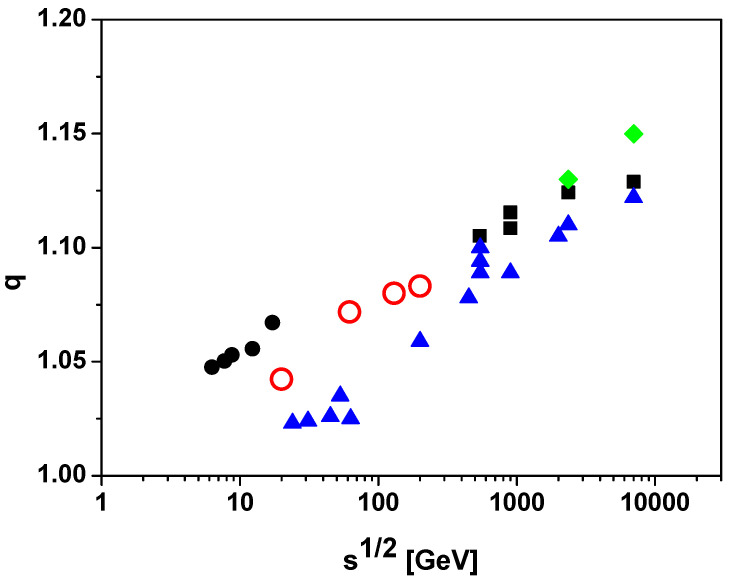
Dependence on $\sqrt{s}$ of the nonextensivity parameter *q* inferred from distinct observables. Filled symbols correspond to *q* extracted from analyses of $f(p_{T})$: a compilation of p+p results (triangles: [28]; diamonds: [29,30]; filled circles: [31]; squares: [32]). Open circles denote *q* inferred from event-by-event fluctuations of $\bar{p_{T}}$.

Using $\omega_{\bar{p_{T}}}^{2}=Var\left(\bar{p_{T}}\right)/{\langle \bar{p_{T}}\rangle }^{2}$ reported by STAR [27], we estimate

(17) $$q-1=\omega_{\bar{p_{T}}}^{2}\;N_{coll},$$

taking $N_{coll}$ evaluated with thickness function $T_{AB}$ = 17 mb^−1^. Figure 1 compares the resulting *q* values with those obtained from independent extractions based on transverse-momentum spectra [28,29,30,31,32].

## 4. Conclusions and Outlook

This study has clarified that the quasi-power-law structures observed in multiparticle production can be understood within two conceptually distinct fluctuation pictures: (i) variations in the effective scale parameter across events, and (ii) fluctuations occurring within each individual event, the latter being operationally equivalent to a nonextensive statistical interpretation. We have further shown that any fluctuation mechanism that produces Tsallis-type multiparticle spectra necessarily generates characteristic correlations among particles in the produced ensemble. On this basis, we proposed an experimental discriminator between intra-event and inter-event variability that exploits eventwise fluctuations of the mean transverse momentum.

Existing RHIC measurements in nucleus–nucleus collisions [27] already indicate $\omega_{\bar{p_{T}}}^{2}>0$, which is consistent with a substantial contribution from fluctuations associated with the nucleon–nucleon sub-collision structure. At present, however, the illustrative estimates summarized in Figure 1 remain insufficient for a definitive interpretation, and a more systematic quantitative analysis is required. In particular, event-by-event measurements of mean-$p_{T}$ fluctuations in proton–proton interactions, where uncertainties related to modeling $N_{coll}$ are absent, would provide a cleaner benchmark and are expected to enable substantially sharper conclusions. Recent measurements carried out in experiments in the Large Hadron Collider on fluctuations in proton–proton collisions open avenues for future investigations in this area [36].

## Data Availability

The original contributions presented in this study are included in the article. Further inquiries can be directed to the corresponding author.
